# Resting-State fMRI Associated with Stop-Signal Task Performance in Healthy Middle-Aged and Elderly People

**DOI:** 10.3389/fpsyg.2017.00766

**Published:** 2017-05-12

**Authors:** Hsing-Hao Lee, Shulan Hsieh

**Affiliations:** ^1^Department of Psychology, National Cheng Kung UniversityTainan, Taiwan; ^2^Institue of Allied Health Sciences, National Cheng Kung UniversityTainan, Taiwan; ^3^Department and Institute of Public Health, National Cheng Kung UniversityTainan, Taiwan

**Keywords:** cluster-analysis, ReHo, fALFF, stop-signal, age

## Abstract

Several brain regions and connectivity networks may be altered as aging occurs. We are interested in investigating if resting-state functional magnetic resonance imaging (RS-fMRI) can also be valid as an indicator of individual differences in association with inhibition performance among aged (including middle-aged) people. Seventy-two healthy adults (40–77 years of age) were recruited. Their RS-fMRI images were acquired and analyzed via two cluster-analysis methods: local synchronization of spontaneous brain activity measured by regional homogeneity (ReHo) and fractional amplitude of low-frequency fluctuations (fALFF) of blood oxygenation level-dependent signals. After the RS-fMRI acquisition, participants were instructed to perform a stop-signal task, in which the stop signal reaction time (SSRT) was calculated based on the horse-race model. Among participants, the ReHo/fALFF and SSRT were correlated with and without partialling-out the effect of age. The results of this study showed that, although aging may alter brain networks, the spontaneous activity of the age-related brain networks can still serve as an effective indicator of individual differences in association with inhibitory performance in healthy middle-aged and elderly people. This is the first study to use both ReHo and fALFF on the same dataset for conjunction analyses showing the relationship between stopping performance and RS-fMRI in the elderly population. The relationship may have practical clinical applications. Based on the overall results, the current study demonstrated that the bilateral inferior frontal gyrus and parts of the default mode network activation were negatively correlated with SSRT, suggesting that they have crucial roles in inhibitory function. However, the pre-supplementary motor area (pre-SMA) and SMA played only a small role during the resting state in association with stopping performance.

## Introduction

Stopping behavior is an important function for an individual in daily activities, such as stopping driving upon seeing an unexpected pedestrian cross the road. Thus, decreasing the adaptive stopping ability may cause severe danger. It has been shown that when people get older, they are more susceptible to distraction (distractibility hypothesis of aging; see Healey et al., 2008 for a review) and disinhibition (inhibition deficit hypothesis of aging; e.g., Hasher and Zacks, 1988; Dempster, 1992; Hasher et al., 1999; Gazzaley and D'Esposto, 2007), resulting in more response intrusions and/or interference from irrelevant stimuli. However, other research indicates that not all older adults are affected by disinhibition, and there might be exceptions either because of individual differences in their brain activity or performance strategies (Hsieh and Fang, 2012; Hsieh and Lin, 2014; Hsieh et al., 2015, 2016). Therefore, research investigating if older adults have generic deficits in inhibition, which to some extent depends upon individual differences in brain activity, is still warranted. In this study we used resting-state functional magnetic resonance imaging (RS-fMRI) in the elderly population. Our main goal was to assess the validity of this resting-state approach, in order to provide a reliable brain-measurement of inhibition in a population for which extensive task-related fMRI testing may not be suitable.

RS-fMRI is a well-known and promising tool to study the relationship between spontaneous brain activity and behavioral performance. Understanding these relationships may have practical clinical applications, especially to infer how well an individual will perform a task if their on-task brain activity is somehow difficult to acquire. Thus, recent studies have investigated the relationship between RS-fMRI and task performance using various cognitive control tasks, including the N-back working memory test (Evers et al., 2012; Sala-Llonch et al., 2012), Stroop task (Evers et al., 2012; Takeuchi et al., 2015), Eriksen flanker task (Mennes et al., 2013), and California verbal learning test (Ystad et al., 2010). In addition, RS-fMRI has also been shown to be correlated to stopping ability (Tian et al., 2012; Hu et al., 2014), which is of main interest in this study. However, these two previous studies were either focused mainly on healthy young adults or biased to sample more healthy young adults across a life-span database, and thus, the association of RS-fMRI with stopping behavior in the middle-aged and elderly is unclear. Therefore, the main purpose of this study is to address this knowledge gap.

Several analytical methods for measuring RS-fMRI have been developed since Biswal et al.'s (1995) pioneering work (for a review, see Zuo and Xing, 2014). These analytical methods can be broadly classified into two categories: one for depicting functional connectivity (FC) between remote brain regions, and one for local FC. The spatial scale for differentiating local FC and remote FC is usually between 10 and 15 mm (e.g., 14 mm used in Sepulcre et al., 2010). The widely used methods in the latter category (local FC) include regional homogeneity (ReHo; Zang et al., 2004), and the amplitude of low-frequency fluctuations (ALFF or fractional ALFF [fALFF]) (Zou et al., 2008). The common rationale for ReHo and f/ALFF methods is that the identification of similar local features of the spontaneous BOLD signal among neighboring voxels within small clusters provides an account of regional functional connectivity. Therefore, ReHo and f/ALFF may be equally useful for exploratory or clinical research because they involve data-driven analyses of the entire brain (whole-brain approach), requiring no *a priori* selection of brain regions of interest (ROIs), which is required for other RS-fMRI methods such as the seed-based approach (Biswal et al., 1997; Cordes et al., 2000; Jiang et al., 2004). However, ReHo and f/ALFF differ in terms of their clustering algorithms (i.e., definition of similarity), as follows: ReHo measures the ***temporal*** synchronization by calculating Kendall's coefficient of concordance (Kendall and Gibbons, 1990) for the time series of a given cluster of neighboring voxels (time-domain analysis), whereas f/ALFF measures the correlation of ***amplitude***/fractional amplitude of spontaneous low-frequency (0.01–0.1 Hz) voxel fluctuations (frequency-domain analysis). Some studies showed strong coupling relationships between ALFF and fALFF (Zou et al., 2008; Zuo et al., 2010), and strong positive correlations between ReHo and ALFF, which suggests that high spontaneous enhanced activity in a given voxel is accompanied by increased synchronization of the surrounding voxels and enhanced amplitude fluctuations of the resting-state blood oxygenation level dependent (BOLD) signals (Yuan et al., 2013; see also Nugent et al., 2015). However, some studies suggested that f/ALFF may be complementary to ReHo, thus suggesting that researchers apply both ReHo and f/ALFF methods to explore which method is more sensitive to local abnormalities and the extent to which they can detect different abnormalities in clinical populations (An et al., 2013; see also Han et al., 2011; Lei et al., 2012; Cui et al., 2014; Premi et al., 2014). Ni et al. (2016) further advocated the advantage of applying these two complementary methods in a study by commenting that: “These two methods are based on different neurophysiology mechanisms…Since the two methods found some changes in common cerebral functional regions, both were adopted to reduce inaccuracies and to provide reliable and comprehensive conclusions” (Ni et al., 2016, p. 1,251).

The aim of this study was to investigate the relationship between stopping performance and spontaneous brain activity in the elderly population, and to assess the feasibility of this approach for future use with older adults for whom extensive task-based fMRI testing may not be suitable. As the brain ages, several brain regions and connectivity networks could be altered in terms of dynamics and location, decreasing the accuracy of ROI- and seed-based analyses. For example, Tomasi and Volkow (2012) reported that aging was associated with pronounced decreases in long-range functional connectivity density in the default mode network (DMN) and dorsal attention network (DAN), and that it was also associated with increases in somatosensory and subcortical networks. Evers et al. (2012) further observed that age-related decreases in functional connectivity in DMN begin in middle-age. Moreover, some aging studies have also shown that the local and remote FC may be modulated by aging with a bias from the remote to local FC in the aged population (Tomasi and Volkow, 2012). Under these circumstances, whole-brain data-driven methods such as ReHo and f/ALFF provide a more reliable approach. These two methods provide different, but complementary neurophysiological measures of regional FC (i.e., time vs. frequency domains). In accordance with the view presented in Ni et al. (2016), here we focus mainly on the overlap between ReHo and fALFF, by means of a conservative conjunction analysis, allowing more reliable and comprehensive conclusions regarding regional FC.

The task used in this study to examine stopping ability is the stop-signal paradigm introduced by Logan and Cowan (1984). In behavioral studies, older adults have often been shown to exhibit longer stopping time, suggesting that there is an age-related inhibitory deficit (e.g., Kramer et al., 1994; Bedard et al., 2002; van de Laar et al., 2011; Kleerekooper et al., 2016). However, other studies found either no age-related decline (e.g., Kray et al., 2009) or only a specific deficit (e.g., Anguera and Gazzaley, 2012). This suggests that aging *per se* may not be the only factor that modulates the inhibitory process. There could be individual differences such as individual brain functions that modulate the inhibitory process. Therefore, in this study, we are particularly interested in investigating whether RS-fMRI is correlated with stop-signal reaction time (SSRT), dependent or independent of aging.

Previous research of ***on-task*** fMRI has shown that the right inferior frontal cortex (rIFC) and (pre-)supplementary motor area ([pre-]SMA) are important brain regions that are associated with successful response inhibition (Li et al., 2006; Chevrier et al., 2007; Chao et al., 2009; Hu et al., 2014). Additional brain areas, such as the superior frontal gyrus (SFG), medial frontal gyrus (medial FG), middle temporal gyrus (MTG), precuneus, and insula have also been reported to be involved in inhibition (Aron and Poldrack, 2006; Li et al., 2006; Ramautar et al., 2006; Chevrier et al., 2007; Erika-Florence et al., 2014). However, RS-fMRI studies of response inhibition among healthy young adults have also shown that ReHo and SSRT correlate significantly in the rIFC (Tian et al., 2012), and that fALFF and SSRT correlate significantly in the pre-SMA/SMA (Hu et al., 2014). As described above, when the brain ages, several brain regions and connectivity networks could be altered, and thus, we are interested in investigating if RS-fMRI is still a valid indicator of individual differences that are associated with inhibition performance among the elderly (including the middle-aged) population. Based on previous research, we expect that there will be a significant correlation between RS-fMRI and SSRT in the rIFC and/or pre-SMA/SMA if aging is not the only factor that modulates the inhibitory process.

## Methods

### Participants

The present study recruited 80 participants from southern Taiwan through advertisement on the internet and on bulletin boards. All participants provided written informed consent, and the study protocol was approved by the Research Ethics Committee of the National Cheng Kung University, Tainan, Taiwan, R.O.C. All participants were paid 1,500 NTD after completion of the experiment. All participants were assessed using the Montreal Cognitive Assessment (MoCA; Nasreddine et al., 2005) and the Beck Depression Inventory II (BDI-II; Beck et al., 1996), and participants with scores lower than 22 on the MoCA or higher than 13 on the BDI-II were excluded during the data analysis. The remaining 72 participants (mean age, 59.38 years, age range 40–77 years, mean education, 13.72 years; 35 males) were all free from current psychological disorders and neurological disease, and they were all right-handed (Table 1).

**Table 1:** **Participant demography and clinical characteristics**.

	**Mean**	**Range**	**SE**
Age	59.38	40–77	1.15
Education	13.72	4–18	0.30
MoCA	26.65	22–30	0.21
BDI-II	4.86	0–13	0.49

*SE, standard error; MoCA, Montreal Cognitive Assessment; BDI-II, Beck Depression Inventory II*.

### Behavioral task: stop-signal task

Participants were seated in a sound-attenuated room, 90 cm away from a computer monitor that presented stimuli. They were instructed to look at the stimulus shown on the monitor's screen, and press the “z” or “/” button corresponding to the target “O” or “X” with their left and right index finger respectively. The screen's background was white and the target stimulus was black. The target stimulus “O” or “X” was presented in the center of the screen lasting for 100 ms (it was 2 cm in size and at a visual angle of 0.64°). An auditory stop signal was presented that had a duration of 300 ms and a frequency of 500 Hz. In the formal experiment, there were 40 stop-trials along with 100 go-trials per block. The stop-signal delay (SSD) varied depending on the participants' response to the stop-trials, and the SSD for each stop-trial was selected from one of two interleaved staircases, each starting with SSD values of 150 and 350 ms. If participants stopped successfully, the SSD would increase 50 ms in the next stop-trial, otherwise there was a decrease of 50 ms in the next stop-trial if they failed to stop (SSD range, 0–800 ms). The staircase procedure ensured that subject's likelihood of stopping converged to 50% chance. The inter-stimulus interval (ISI) varied from 1,300 to 4,800 ms, and the SSRT was calculated by subtracted the median SSD from the median RT of the go trials (Band et al., 2003).

There were two practice blocks before the formal experiment started. In the first practice block, participants were instructed to perform a choice reaction-time task. Participants were instructed to respond to the stimulus as soon and as accurately as possible. There was a “beep” sound in the background and participants were asked to ignore this sound. In the second block of practice, participants were instructed to stop their reaction immediately when they heard the stop signal of a “beep” sound following the stimulus onset. They were told not to slow down their reaction to wait for the stop signal to occur. After the practice, the formal experiment commenced, and all of the settings and rules were the same as for the second practice block. The formal experiment included five blocks (140 trials per block, and 40 of them were stop-trials). The completion time was approximately 30 min, including instruction and practice time.

### fMRI acquisition and processing

MRI images were acquired using a GE MR750 3T scanner (GE Healthcare, Waukesha, WI, USA) in the Mind Research Imaging (MRI) center at the National Cheng Kung University. High-resolution structural images were acquired using fast-SPGR, consisting 166 axial slices (TR/TE/flip angle, 7.6 ms/3.3 ms/12°; field of view (FOV), 22.4 × 22.4 cm^2^; matrices, 224 × 224; slice thickness, 1 mm), and the entire process lasted for 218 s. The resting-state functional images were collected using a gradient-echo planar imaging (EPI) pulse sequence (TR/TE/flip angle, 2,000 ms/30 ms/77°; matrices, 64 × 64; FOV, 22 × 22 cm^2^; slice thickness, 4 mm; voxel size, 3.4375 × 3.4375 × 4 mm). These slices covered the whole brain of each participant, and the scan time was 490 sec ((number of samples + number of dummy scan) × TR = (240+5) × 2 = 490 s) per subject. During the resting-state functional scans, the participants were instructed to remain awake with their eyes open and fixate on the white cross shown on the screen.

#### Image analysis: preprocessing

The imaging data were preprocessed through SPM8 in MATLAB (The MathWorks, Inc., Natick, MA, USA). Functional images underwent slice timing, realignment, and coregistration. In the realignment, the time series of the scan was aligned to the first image of the session to correct the head motion. T1 images were then co-registered to participant's own EPI images and normalized (Bonding Box: −100, −130, −80; 100, 100, 110) to the Montreal Neurological Institute (MNI) standard space that was defined by a template T1-weighted image and resliced using a voxel size of 2 × 2 × 2 mm^3^ to agree with the gray matter probability maps, and spatial smoothing was performed with a 6-mm full-width at half-maximum Gaussian kernel. Finally, every voxel was band-pass filtered (0.01–0.08 Hz) to reduce the noise of high and low frequency fluctuations.

#### Resting-state image analysis

A two-step analysis of the resting-state data was performed as follows: (1) the resting-state data underwent two different kinds of analysis: ReHo and fALFF; and (2) the results of ReHo and fALFF were then correlated with the behavioral data (i.e., SSRT; see below for details). In addition, the correlations between ReHo and SSRT, as well as between fALFF and SSRT also added age as a covariate to re-examine the correlations.

#### ReHo

ReHo analysis was performed on a voxel-by-voxel basis by calculating Kendall's coefficient of concordance (KCC) (Kendall and Gibbons, 1990) of the time series in a given cluster of the neighboring 27 voxels. ReHo analysis is based on the hypothesis that significant brain activity would occur in a cluster rather than a single voxel. The ReHo value was assigned to the central voxel, which can represent the similarity of several time series (see Zang et al., 2004 for details).

##### fALFF

The filtered resting-state data were transformed into the frequency domain using fast Fourier transform (FFT), and the power was calculated using the square root of the spectrum. Differing from the ALFF method, BOLD frequencies within 0.01–0.08 Hz were divided by the entire frequency range (0.01–0.25 Hz; unfiltered signals) at each voxel to obtain the fALFF value, which is less sensitive to physiological noise than the ALFF method (see Zou et al., 2008 for details).

##### Relationship between resting-state and behavioral performance: ReHo-SSRT and fALFF-SSRT correlations

The ReHo and SSRT correlation was calculated using the REST in-house function (Statistical Analysis, REST Correlation Analysis). The critical correlation value was set at 0.31 under the criteria of α = 0.005 (degrees of freedom, 70). We calculated the ReHo and SSRT correlation, as well as the ReHo and SSRT correlation partialled-out for the effect of age, using age as a covariate in the correlation analysis function in REST. We performed the same processes for the fALFF and SSRT correlation analysis. We inspected the significant areas using the REST Viewer. The cluster threshold was set at *p* < 0.005, which was corrected using AlphaSim. The AlphaSim correction is a method to provide a reasonable significance level while avoiding false-positive activation during analysis. This approach went through the iteration of random image generation, Gaussian filtering, scaling and thresholding, mask application, and cluster identification. It estimates the overall required significance level for multiple combinations of probability threshold and cluster size threshold (Ward, 2000). Other studies also used this correction approach to probe into aging and resting-state fMRI issues (e.g., Wu M. et al., 2011; Wu J. T. et al., 2011).

##### Conjunction analysis of ReHo-SSRT and fALFF-SSRT correlation maps

We used the minimal *t*-statistic approach rationale (Nichols et al., 2005) to calculate the conjunction of ReHo-SSRT and fALFF-SSRT full-/partial-correlation maps. Instead of the minimal *t*-value used by Nichols' research group, we used the maximum *r*-value as the conjunction calculation index because we were interested in the negative correlation between the resting-state brain network and SSRT. We created a new brain map that extracted the maximum of the negative r-value in each voxel of ReHo-SSRT or fALFF-SSRT full-/partial-correlation map, which were not corrected or thresholded. This map underwent AlphaSim correction again and the surviving significant areas indicated the overlap areas that were significantly correlated with SSRT under both ReHo and fALFF analyses. All the calculations were performed using the MATLAB in-house code.

## Results

### Behavior results

The behavioral performance of the go and stop (stop-success and stop-failure) trials in the stop-signal task is summarized in Table 2.

**Table 2:** **Behavioral data**.

		**RT (ms)**	**Choice Error (%)**	**Omission (%)**
Go trials		677.21 (17.05)	2.07 (0.37)	11.24 (1.39)
	**% of Inhibit**	**RT (ms)**	**SSD (ms)**	**SSRT (ms)**
Stop-success trials	51.34 (1.41)	—	352.96 (19.69)	302.19 (20.50)
Stop-failure trials	—	597.13 (15.10)	386.70 (21.69)	—

*(standard error [SE] between parentheses): (1) Mean reaction time (RT), percentage of choice error (%) and omission (%) associated with go-trials; (2) mean percentage of inhibition (%), stop-signal delay (SSD; ms) and stop-signal RT (SSRT; ms) associated with stop-success trials; mean RT and stop-signal delay (SSD; ms) associated with stop-failure trials*.

#### Go-trial performance

The mean accuracy of the correct go-trials was 86.69 ± 1.58%, and the RT was 677.21 ± 17.05 ms. The Pearson correlation between go-trials' RT and age was not significant (*r* = 0.05, *p* = 0.66), and the correlation between go-trials' accuracy and age was also not significant (*r* = −0.10, *p* = 0.43).

#### Stop-trial performance

The stop inhibition rate (stop success rate) was 51.34 ± 1.41%, which was close to the 50% aimed at by the staircase algorithm. The average SSRT was 302.19 ± 20.50 ms. The correlation between SSRT and age did not reach significance (*r* = 0.22, *p* = 0.07; Cohen's *d* = 0.45), suggesting that stopping performance did not change significantly with age, at least in the age range of 40–77 years. In addition, the correlation between SSRT and MoCA (*r* = −0.19, *p* = 0.12), and that between SSRT and BDI (*r* = −0.02, *p* = 0.90) did not reach significance. No significant correlation was found between the mean RT of the correct go-trials and the SSRT (*r* = −0.04, *p* = 0.75), which is consistent with the horse-race model that assumes the independence of the process between go-trials and stop-trials.

### fMRI results

#### ReHo and SSRT correlations

**Figure 1:**
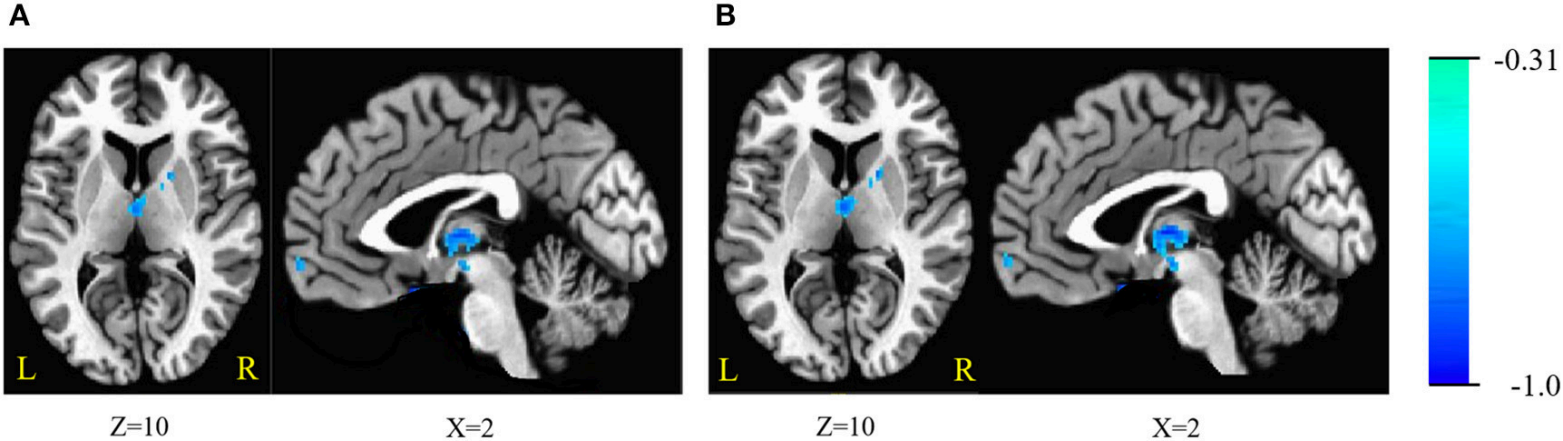
**(A)** Brain areas of regional homogeneity (ReHo) are negatively correlated with stop-signal reaction time (SSRT) across participants; **(B)** with age as a covariate. Blue indicates negative correlations. The threshold was set at *p* < 0.005 under AlphaSim correction. The number below the images refer to the z coordinates (axial view), or x coordinates (sagittal view).

The ReHo was negatively correlated with SSRT in the bilateral cerebellum, bilateral superior frontal gyrus (SFG), medial frontal gyrus, bilateral inferior frontal gyrus (IFG), bilateral inferior temporal gyrus (ITG), bilateral fusiform gyrus (FG), parahippocampal gyrus (PHG), thalamus, lentiform nucleus, left putamen, right caudate, left insula, and pons (cluster *p* < 0.005 corrected for AlphaSim). Because we recruited participants with a wide range of ages, we partialled-out the effect of age during the ReHo and SSRT correlation analyses to clarify if age plays a crucial role in the correlation. We found that the right SFG, left IFG, putamen and insula did not survive after the partialling-out process. The results are summarized in Tables 3, 4, and in Figure 1.

**Table 3:** **Brain areas of regional homogeneity (ReHo) are negatively correlated with stop-signal reaction time (SSRT) across participants**.

**Region**	**Cluster size(voxels)**	**Peak MNI coordinates**			***r***
		***x***	***y***	***z***	
Right cerebellum	39	44	−72	−54	−0.45
	42	22	−90	−28	−0.39
Left cerebellum	39	−48	−56	−50	−0.51
Right SFG	25	14	46	−22	−0.54
Left SFG	36	−18	58	14	−0.49
Medial frontal gyrus	26	2	58	−6	−0.45
Right IFG	23	40	20	−14	−0.48
Left IFG	17	0	8	−16	−0.56
Right ITG/Right FG/PHG	157/149/44	72	0	−34	−0.5
Left ITG/FG	71/37	−60	−6	−30	−0.49
Left ITG/Left FG	99/48	−50	−44	−28	−0.55
Thalamus	151	0	−12	8	−0.53
Lentiform Nucleus/Left putamen	30/28	−24	6	−2	−0.41
Right caudate	50	18	2	14	−0.46
Left insula	23	−38	12	−10	−0.47
Pons	70	4	−18	−38	−0.46

*SFG, superior frontal gyrus; IFG, inferior frontal gyrus; ITG, inferior temporal gyrus; FG, fusiform gyrus; PHG, parahippocampal gyrus*.

**Table 4:** **Brain areas of regional homogeneity (ReHo) are negatively correlated with stop-signal reaction time (SSRT) across participants, with age as a covariate**.

**Region**	**Cluster size(voxels)**	**Peak MNI coordinates**			***r***
		***x***	***y***	***z***	
Right cerebellum	27	48	−44	−32	−0.46
Left cerebellum	29	−48	−52	−50	−0.48
Left SFG	42	−18	58	14	−0.49
Medial frontal gyrus	26	2	58	-6	−0.46
Right IFG	20	40	20	−14	−0.45
Right ITG/Right FG/PHG/Pons	200/187/67/26	12	−12	−28	−0.56
Left ITG/Left FG	182/51	−50	−44	−28	−0.54
Thalamus	173	0	−12	8	−0.57
Lentiform Nucleus/Putamen	45/25	−24	6	−2	−0.44
Right caudate	41	18	2	14	−0.47

*SFG, superior frontal gyrus; IFG, inferior frontal gyrus; ITG, inferior temporal gyrus; FG, fusiform gyrus; PHG, parahippocampal gyrus*.

#### fALFF and SSRT correlations

**Figure 2:**
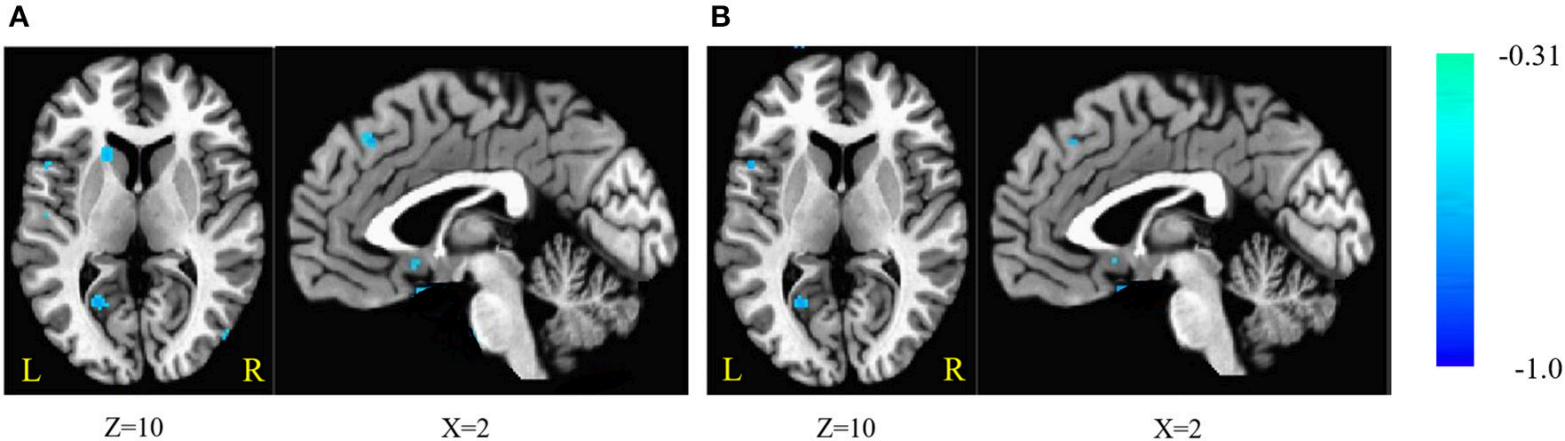
**(A)** Brain areas of fractional amplitude of low-frequency fluctuations (fALFF) are negatively correlated with stop-signal reaction time (SSRT) across participants; **(B)** with age as a covariate. Blue indicates negative correlations. The threshold was set at *p* < 0.005 under AlphaSim correction. The number below the images refer to the z coordinates (axial view), or x coordinates (sagittal view).

The fALFF was negatively correlated with SSRT in the bilateral cerebellum, left SFG, right middle frontal gyrus, left IFG, right superior temporal gyrus (STG), right MTG, bilateral ITG, left superior parietal lobule (SPL), right inferior parietal lobule (IPL), right post-central gyrus, left pre-central gyrus, post-central gyrus, left supramarginal gyrus (SMG), right SMA, medial frontal gyrus, right middle occipital gyrus (MOG), bilateral FG, posterior cingulate cortex (PCC), parahippocampal gyrus (PHG), left caudate, left putamen, and pons (cluster *p* < 0.005 corrected for AlphaSim). The left SPL, right SMA, and pons did not survive after we partialled-out the effect of age. The results are summarized in Tables 5, 6, and in Figure 2.

**Table 5:** **Brain areas of fractional amplitude of low-frequency fluctuations (fALFF) are negatively correlated with stop-signal reaction time (SSRT) across participants**.

**Region**	**Cluster size(voxels)**	**Peak MNI coordinates**			***r***
		***x***	***y***	***z***	
Right cerebellum	66	44	−72	−54	−0.46
Left cerebellum/Left ITG/ Left FG/PHG	181/112/81/77	−50	−44	−28	−0.6
Left SFG/Medial frontal gyrus	62/23	−8	30	54	−0.51
Right MFG	28	30	−8	60	−0.41
Left IFG/Left pre-central gyrus	78/21	−36	6	20	−0.45
Right STG	31	38	14	−26	−0.46
Right MTG/Right MOG	63/50	44	−92	−4	−0.42
Right ITG/Right FG PHG/Pons	321/182/88/94	60	32	−38	−0.55
Left ITG/FG	85/49	−70	−2	−32	−0.52
Left SPL	27	−32	−52	64	−0.42
Right post-central gyrus/Right IPL	37/24	30	−44	52	−0.47
Post-central gyrus/Left SMG	41/35	−54	−24	18	−0.43
Right SMA/Medial frontal gyrus	32/24	12	−14	74	−0.43
PCC	38	−22	−66	6	−0.46
Left caudate/Left putamen	57/51	−10	8	−2	−0.44

*SFG, superior frontal gyrus; MFG, middle frontal gyrus; IFG, inferior frontal gyrus; STG, superior frontal gyrus; MTG, middle temporal gyrus; ITG, inferior temporal gyrus; SPL, superior parietal lobule; IPL, intra-parietal lobule; SMA, supplementary motor area; MOG, middle occipital gyrus; FG, fusiform gyrus; PCC, posterior cingulate cortex; PHG, parahippocampal gyrus*.

**Table 6:** **Brain areas of fractional amplitude of low-frequency fluctuations (fALFF) are negatively correlated with stop-signal reaction time (SSRT) across participants, with age as a covariate**.

**Region**	**Cluster size(voxels)**	**peak MNI coordinates**			***r***
		***x***	***y***	***Z***	
Left cerebellum/Left ITG/Left FG/PHG	163/103/81/77	−50	−44	−28	−0.58
Right cerebellum	93	36	−92	−16	−0.41
Left SFG/Medial frontal gyrus	65/20	−8	30	54	−0.48
MFG/Right pre-central gyrus	57/33	30	−8	58	−0.41
Left IFG/Left pre-central gyrus	109/21	−52	10	12	−0.46
Right STG	26	38	14	−26	−0.43
Right ITG/Right FG/PHG/Right MTG	312/245/90/64	42	−10	−32	−0.52
Left ITG	103	−50	−44	−28	−0.58
Left ITG/FG	83/50	−70	−2	−32	−0.5
Right post-central gyrus/Right IPL	26/22	30	−42	52	−0.46
Post-central gyrus/Left SMG	23/18	−54	−24	18	−0.42
Right MOG	27	44	−92	−4	−0.4
PCC	31	−22	−66	6	−0.46
Left caudate/Left putamen	23/14	−10	8	−2	−0.41

*SFG, superior frontal gyrus; MFG, middle frontal gyrus; IFG, inferior frontal gyrus; STG, superior temporal gyrus; MTG, middle temporal gyrus; ITG, inferior temporal gyrus; IPL, intra-parietal lobule; SMG, supramarginal gyrus; MOG, middle occipital gyrus; FG, fusiform gyrus; PCC, posterior cingulate cortex; PHG, parahippocampal gyrus*.

#### Overlap between ReHo-SSRT and fALFF-SSRT correlations

**Figure 3:**
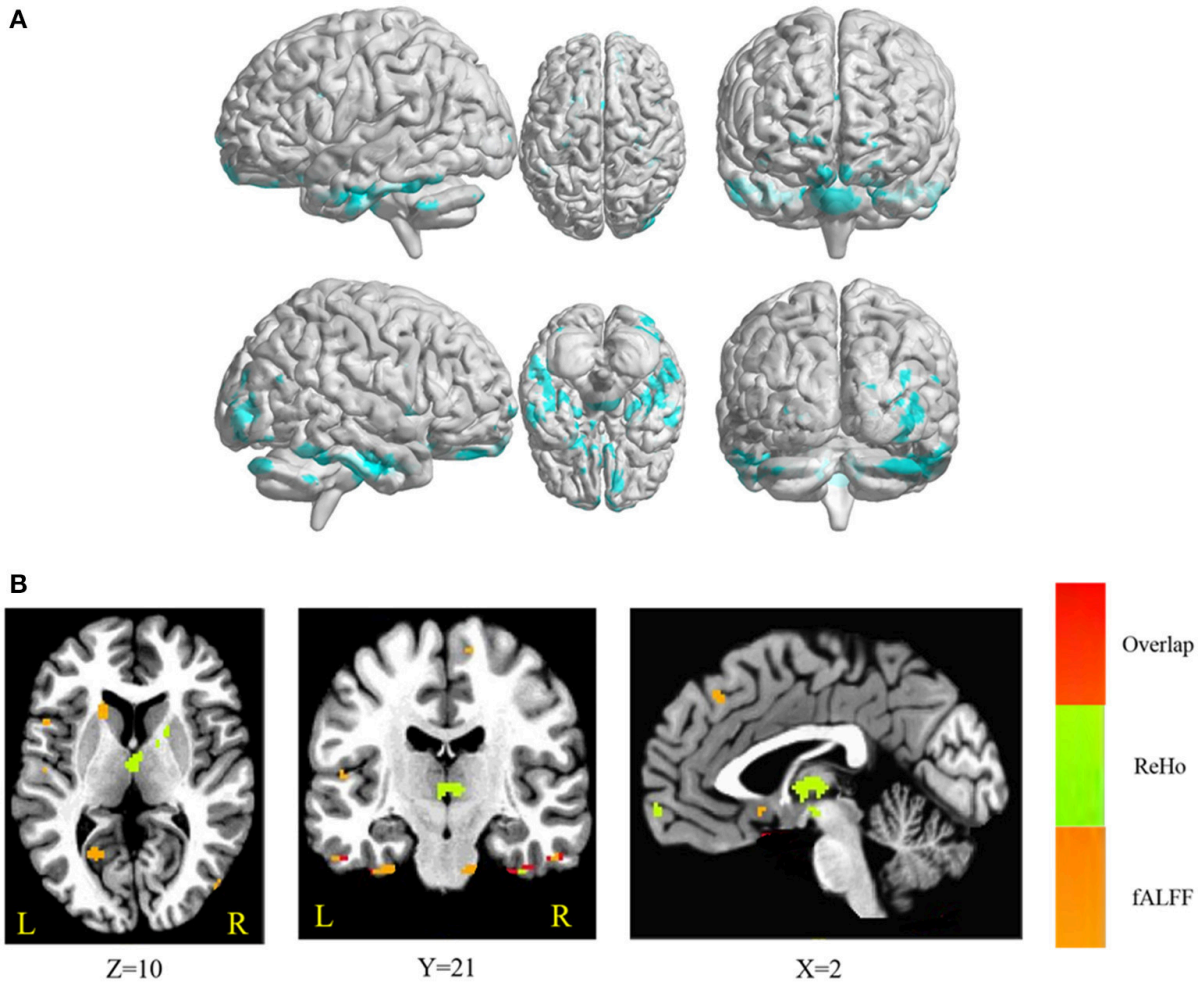
**(A)** Overlapping brain areas between the two sets of correlations via a conjunction analysis: (1) correlation of regional homogeneity (ReHo) and stop-signal reaction time (SSRT); (2) correlation of fractional amplitude of low-frequency fluctuations (fALFF) and SSRT. Blue indicates negative correlations. The threshold was set at *p* < 0.005 under AlphaSim correction; **(B)** Overlap of ReHo-SSRT and fALFF-SSRT correlation maps. The number below the images refer to the z coordinates (axial view), y coordinates (coronal view), or x coordinates (sagittal view). Regions in red show significance in both ReHo-SSRT (in green) and fALFF-SSRT (in orange) correlation maps.

**Figure 4:**
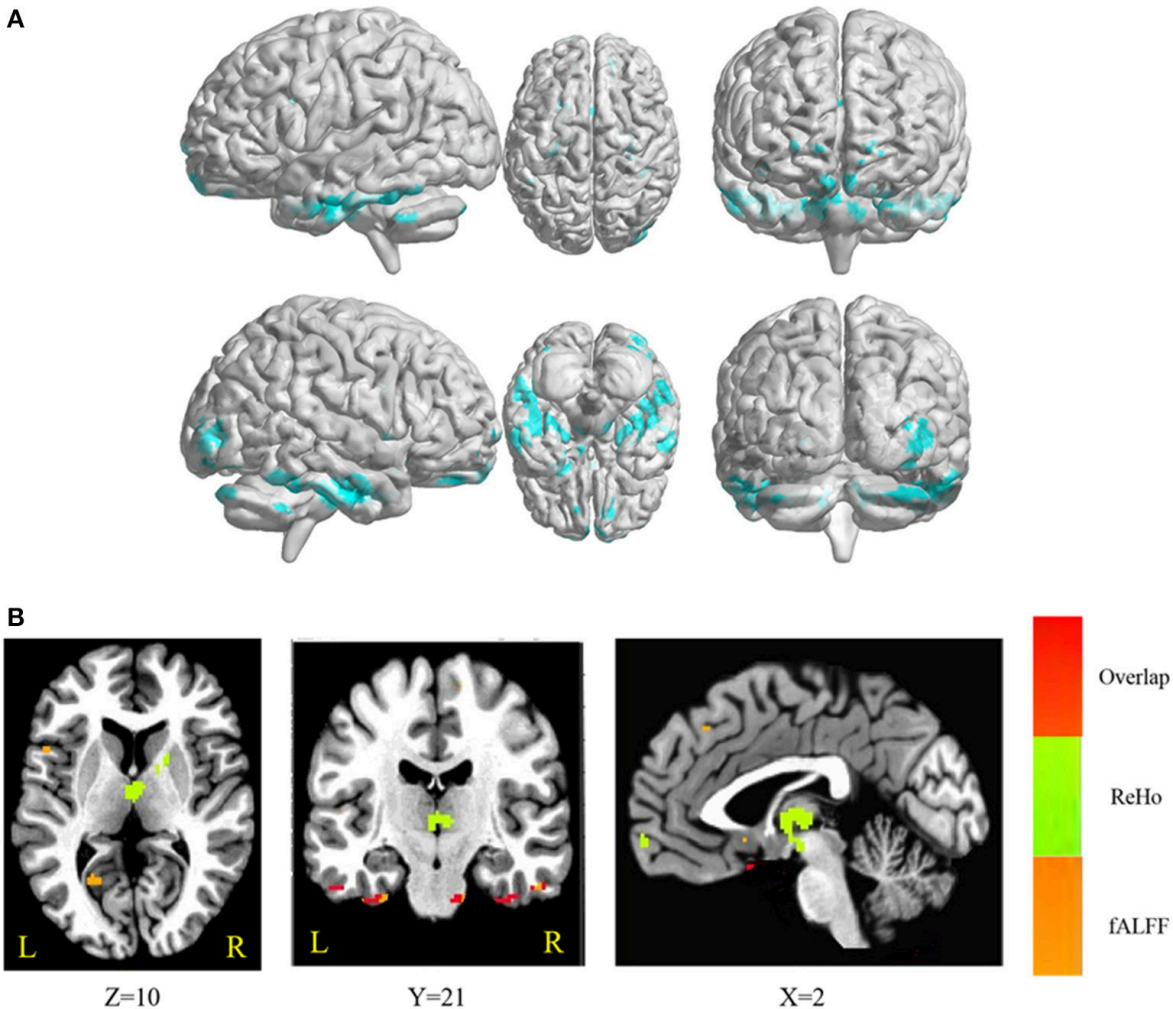
**(A)** Overlapping brain areas between the two sets of correlations via a conjunction analysis: (1) partial correlation of regional homogeneity (ReHo) and stop-signal reaction time (SSRT), with age as a covariate; (2) partial correlation of fractional amplitude of low-frequency fluctuations (fALFF) and SSRT, with age as a covariate. Blue indicates negative correlations. The threshold was set at *p* < 0.005 under AlphaSim correction; **(B)** Overlap of ReHo-SSRT and fALFF-SSRT partial correlation maps, with age as a covariate. The number below the images refer to the z coordinates (axial view), y coordinates (coronal view), or x coordinates (sagittal view). Regions in red show significance in both ReHo-SSRT (in green) and fALFF-SSRT (in orange) partial correlation maps.

The conjunction analysis revealed that bilateral cerebellum, pons, bilateral ITG, bilateral FG, PHG, left IFG, and medial frontal gyrus were found in the overlapping areas of ReHo-SSRT and fALFF-SSRT full-correlations. Overlaps between ReHo-SSRT and fALFF-SSRT partial-correlations (i.e., partialling-out the effect of age) were found in bilateral cerebellum, bilateral ITG, right MTG, bilateral FG, bilateral, PHG, left STG, and left IFG. The results are summarized in Tables 7, 8, and in Figures 3, 4.

**Table 7:** **Overlapping brain areas between the two sets of correlations via a conjunction analysis: (1) correlation of regional homogeneity (ReHo) and stop-signal reaction time (SSRT); (2) correlation of fractional amplitude of low-frequency fluctuations (fALFF) and stop-signal reaction time (SSRT)**.

**Region**	**Cluster size (voxels)**	**Peak MNI coordinates**			***r***
		***x***	***Y***	***Z***	
Right cerebellum	37	44	−72	−54	−0.45
Right cerebellum	31	48	−44	−32	−0.48
Right cerebellum	42	36	−84	−30	−0.37
Right cerebellum	29	56	−62	−28	−0.49
Left cerebellum	33	−48	−52	−50	−0.5
Pons	66	4	−18	−38	−0.45
Lef ITG	7	−72	−4	−30	−0.49
Right ITG/Right FG/Right PHG/Right MTG	153/142/42/31	72	0	−34	−0.5
Left ITG/Left cerebellum/Left FG	98/61/48	−50	−44	−28	−0.55
Left ITG/Left FG	60/35	−60	−6	−30	−0.44
Left PHG/Left IFG	22/15	−20	2	−30	−0.48
Medial frontal gyrus	4	2	64	−20	−0.45

*ITG, inferior temporal gyrus; FG, fusiform gyrus; PHG, parahippocampal gyrus; MTG, middle temporal gyrus; IFG, inferior frontal gyrus*.

**Table 8:** **Overlapping brain areas between the two sets of correlations via a conjunction analysis: (1) Partial correlation of regional homogeneity (ReHo) and stop-signal reaction time (SSRT) with age as a covariate; (2) Partial correlation of fractional amplitude of low-frequency fluctuations (fALFF) and stop-signal reaction time (SSRT) with age as a covariate**.

**Region**	**Cluster size (voxels)**	**Peak MNI coordinates**			***r***
		**x**	**y**	**z**	
Right cerebellum	29	48	−44	−32	−0.46
Left cerebellum	27	−48	−52	−50	−0.47
Right ITG/Right FG/Right PHG/Right MTG	198/185/66/40	72	−2	−34	−0.49
Left ITG/Left cerebellum/Left PHG/Left FG	95/60/53/53	−50	−44	−28	−0.54
Left PHG/Left STG/Left IFG	215/9/9	−20	2	−30	−0.48
Left ITG/Left FG	72/43	−60	−6	−30	−0.45

*ITG, inferior temporal gyrus; FG, fusiform gyrus; PHG, parahippocampal gyrus; MTG, middle temporal gyrus. STG, superior temporal gyrus; IFG, inferior frontal gyrus*.

## Discussion

This study aimed to examine whether spontaneous brain activity evaluated by resting-state fMRI can be used as an indicator of individual differences in association with inhibitory performance even in healthy middle-aged and aged people. We used two local FC analysis methods (ReHo and fALFF) to evaluate the relationship between spontaneous brain connectivity and stopping performance as reflected on SSRT, and we focused on overlapping regions measured across the two methods to provide more conservative results (Ni et al., 2016). The current behavioral results showed that SSRT was not prolonged as a function of age, suggesting that there was no age-related reduction in stopping ability, which was consistent with Kray et al.'s (2009) findings. Yet, the null finding may be simply a result of a reduction in stopping ability that reached a plateau around middle age, because the current study recruited only elderly people. Alternatively, since there seemed to be a “statistical trend” (*p* = 0.07) and the medium effect size (Cohen's *d* = 0.45) seemed to suggest that taking a larger sample size might bring this effect into statistical significance. However, this issue is beyond the scope of the current study, because we focused only on elder populations.

We investigated if RS-fMRI is an effective indicator in association with stopping ability in the elderly. The current RS-fMRI results showed that the FC correlation results of the two local FC analysis methods, i.e., ReHo and fALFF with SSRT, overlapped in several brain regions (Tables 7, 8). These results can be summarized into four main features: the activities during the resting state of (1) some parts of the DMN; and (2) the left IFG and bilateral FG were involved; however, (3) the pre-SMA/SMA was not involved in association with stopping performance; and (4) the correlations between the RS-fMRI and stopping performance that we observed were negative rather than positive. The DMN is an interconnected and anatomically defined set of brain regions, consisting of some functional hubs including the posterior cingulate cortex (PCC)/precuneus, medial prefrontal cortex (mPFC), hippocampus, and angular gyrus. The DMN has been shown to deactivate during external goal-oriented tasks such as visual attention or cognitive working memory tasks but it activates during the resting state, thus, leading some researchers to label the network as the task-negative network (e.g., Raichle et al., 2001; Greicius et al., 2003). Its activities have been hypothesized to potentially influence goal-directed behavior and/or mental effort during cognitive tasks (Weissman et al., 2006; Li et al., 2007), self-referential thinking, emotional processing, and recalling memories. The current findings indicate that the medial frontal gyrus and PHG, which are parts of the DMN, were negatively correlated with SSRT, suggesting that DMN can also serve as an effective indicator to associate with stopping performance even in the middle-aged and aged population. Thus, a main contribution of the current study is to provide new evidence showing that the DMN is also involved and associated with stopping behavior.

Secondly, the current study also shows that the left IFG can serve as an indicator that is associated with stopping performance. The right IFG is well-known to be important for successful response inhibition (for reviews, see Aron et al., 2004; Verbruggen and Logan, 2009). Although the current study observed that the left IFG, rather than the right IFG, was associated with the forthcoming successful inhibition, some studies have also reported bilateral IFC activations (Bunge et al., 2002; Li et al., 2006; Tian et al., 2012) or left IFG activation (Swick et al., 2008), as we showed in this study. Therefore, the current results suggest that the left IFG may also play an important role in response inhibition.

Thirdly, in the current study, pre-SMA was not found to be associated with SSRT performance. The current results seem to be inconsistent with those of other studies, such as those by Chao et al. (2009), Chevrier et al. (2007), Li et al. (2006), and Hu et al. (2014). However, these previous studies mostly investigated the relationship between the pre-SMA and inhibition performance during the on-task period, rather than the pre-task resting period, which we investigated in this study. Therefore, the discrepancy may be attributed to the role of pre-SMA in reactive motor inhibition, rather than the attentional processing of the stop-signal that was modulated by the IFG. Thus, the current results provide indirect evidence showing that the pre-SMA is involved in reactive inhibition, whereas the IFG is involved in the attentional processing of the stop-signal (see also Duann et al., 2009).

Finally, the current findings appear to be consistent with the reports by Tian et al. (2012) showing significant correlations between SSRT and the ReHo of the ITG, STG, IFG, and medial frontal gyrus, but there is one main difference. This difference is that, while Tian et al. (2012) observed positive correlations between SSRT and the IFG, and between SSRT and the DMN (MPFC, IPL, precuneus), our results showed negative correlations. A major difference between Tian et al.'s (2012) results and the current results is in participants' age. This suggests that for younger adults, the correlation between the IFG and SSRT is positive, as shown by Tian et al. (2012), but for middle-aged and aged adults, the correlation becomes negative, as shown in the current study. Another study by Hu et al. (2014) also addressed the correlation between resting-state fMRI and SSRT, but they showed a different pattern with a negative correlation between SSRT and the fALFF in the pre-SMA/SMA. In Hu et al.'s (2014) study, participants' age varied widely, from 18 to 72 years (with 63 out of 111 healthy participants in the age range of 20–29 years, and 18 out of 111 healthy participants in the age range of 30–39 years). A potential hypothesis to explain the discrepancies between the previous findings of positive correlations and the current findings of negative correlations is a shift from remote to local connectivity during aging, which results in a polarity reversal for the correlations between RS-fMRI and SSRT.

To clarify if the current findings on the correlation between RS-fMRI and SSRT were modulated by age, we used age as a covariate while computing the correlations between ReHo/fALFF and SSRT, to partial-out the effect of age. The results showed that only the medial frontal gyrus was excluded and the left STG was additionally included, whereas the bilateral cerebellum, pons, bilateral ITG, right MTG, bilateral FG, bilateral PHG, and left IFG maintained their significantly negative correlation with SSRT. Therefore, the current findings regarding the relationship between resting state activities of these brain regions and stop processing cannot solely be attributed to the effect of age. Our age range included participants in the age range of 40–77 years (mainly middle-aged and aged healthy participants), because this study mainly focused on the elder population. Whether or not this might therefore underestimate the effect of age is beyond the current research scope and further research is required to address this issue. Finally, these results suggest that RS-fMRI may be a viable method to assess inhibition in an older population, in which it is often not feasible perform extensive task-based fMRI testing.

## Conclusions

The results of this study showed that although aging may alter brain networks, the spontaneous activity of the age-related brain networks can still serve as an effective indicator of individual differences in association with inhibitory performance in healthy middle-aged and elderly people. The current findings have two major contributions: (1) clinical application: one may infer the stopping efficacy based on the resting-state neuroimaging for individuals who have difficulties completing the task; and (2) methodological conservativeness: this is the first study to use both ReHo and fALFF on the same dataset for conjunction analyses to reduce inferring inaccuracies and provide reliable and comprehensive conclusions regarding regional functional connectivity.

## Author contributions

HL collected the data, analyzed the data, and drafted some parts of the manuscript. SH initiated the research idea, applied for the funding, designed the task, supervised data analyses, drafted and revised the manuscript.

## Funding

This work was supported by the Ministry of Science Technology (MOST) of the Republic of China, Taiwan for financially supporting this research (Contract No. 104-2410-H-006-021-MY2).

### Conflict of interest statement

The authors declare that the research was conducted in the absence of any commercial or financial relationships that could be construed as a potential conflict of interest.
